# Model-Based Prediction of Acid Suppression and Proposal of a New Dosing Regimen of Fexuprazan in Humans

**DOI:** 10.3390/ph15060709

**Published:** 2022-06-03

**Authors:** Min-Soo Kim, Nora Lee, Areum Lee, Yoon-Jee Chae, Suk-Jae Chung, Kyeong-Ryoon Lee

**Affiliations:** 1 College of Pharmacy, Seoul National University, Seoul 08826, Korea; misol@snu.ac.kr; 2 RIKEN Innovation Center, Research Cluster for Innovation, RIKEN, Yokohama 230-0045, Kanagawa, Japan; nora37@gmail.com; 3 Division of Biopharmaceutics, College of Pharmacy, Kyung Hee University, Seoul 02447, Korea; pharmareum@gmail.com; 4 College of Pharmacy, Woosuk University, Wanju-gun 55338, Korea; yjchae@woosuk.ac.kr; 5 Research Institute of Pharmaceutical Sciences, Seoul National University, Seoul 08826, Korea; 6 Laboratory Animal Resource Center, Korea Research Institute of Bioscience and Biotechnology, Cheongju 28116, Korea; 7 Department of Bioscience, University of Science and Technology, Daejeon 34113, Korea

**Keywords:** DWP14012, fexuprazan, model-informed drug development, pharmacodynamic modeling, pharmacokinetic modeling, potassium-competitive acid blocker

## Abstract

Fexuprazan is a potassium-competitive acid blocker (P-CAB). The compounds in this newly developed drug family suppress intragastric acidity. As there are already other acid-suppressing drugs on the market, such as H_2_ antagonists and proton pump inhibitors (PPIs), it would be informative to compare the biological effects of fexuprazan against another approved drug with the same indication. The drug concentration predicted by the pharmacokinetic (PK) model could serve as an input function for a pharmacodynamic (PD) model. The apparent pharmacokinetics of fexuprazan could be described by a simpler model. However, a physiologically based pharmacokinetic (PBPK) model was developed in a previous study. A one-compartment model was also proposed in the present study. Both the newly suggested model and the previously validated PBPK model were used as input functions of the PD models. Our simulation revealed that the effects of fexuprazan could be effectively simulated by the proposed PK–PD models. A PK–PD model was also proposed for the oral administration of the PPI reference drug esomeprazole. A model-based analysis was then performed for intragastric pH using several dosing methods. The expected pH could be predicted for both drugs under several dosing regimens using the proposed PK–PD models.

## 1. Introduction

Fexuprazan is a recently approved potassium-competitive acid blocker (P-CAB) in the Republic of Korea (Fexuclue Tablet 40 mg) [1,2,3,4,5,6]. P-CAB drugs are competitive, reversible H^+^/K^+^ ATPase inhibitors [1,2,3] which can increase gastric pH. Maintaining a pH > 4 was considered one of the best approaches to alleviating esophageal mucosal injury in gastroesophageal reflux disease (GERD) patients [7]. Several drugs are approved to suppress intragastric acidity. Comparing the biological effects will give us insights into effective drug therapy, such as a new dosing regimen. Before the development of P-CAB drugs, other acid suppressors such as H_2_ receptor antagonists and proton pump inhibitors (PPIs) were widely used to treat GERD, gastric ulcer, and *H. pylori* infection [8,9,10,11].

P-CABs and PPIs both inhibit H^+^/K^+^ ATPase [11]. Clinical trials revealed that a daily dose of 40 mg of fexuprazan had a similar pH-raising efficacy as 40 mg of the PPI drug esomeprazole at the ‘once daily’ interval [1]. The similarity in the mechanism implies that relatively new P-CABs will have similar indications to PPIs. It was expected that the fexuprazan dosing regimen could be extended to the twice daily regimen, which has been used for esomeprazole and the other PPIs [9], but there was no quantitative and dynamic prediction before. Integrated pharmacokinetic (PK) and pharmacodynamic (PD) prediction will be useful for the question [12]. The concentration predicted by a PK model can be incorporated into a PD model. PK analysis was not enough for this study, and PD prediction was performed to compare the effects of two compounds (i.e., fexuprazan and esomeprazole) and to propose a new dosing regimen for fexuprazan.

This model-based approach is useful for designing dosage regimens and studying formulations and drug–drug interaction mechanisms in the future [3,13,14,15,16]. Here, we performed non-compartmental and compartmental analyses, including the PBPK model, to predict the PK profiles for fexuprazan in the human plasma and stomach [17].

We also used PBPK modeling to predict the esomeprazole concentration profiles in human plasma. Representative PD models include simple direct effect, biophase distribution, slow receptor-binding, and indirect response models [18,19]. The simple direct effect and indirect response models were adopted here to describe the pharmacodynamic effects of fexuprazan. There was a time interval between the maximum plasma concentration (a pharmacokinetic indicator) and the maximum pH (a pharmacodynamic indicator). Hence, an indirect response model was linked using the predicted plasma concentrations for fexuprazan and esomeprazole. However, the simple direct effect model was also applied to predict gastric pH in response to fexuprazan using the stomach (target site) concentration obtained by PBPK modeling. Four and one PK–PD models were proposed for fexuprazan and esomeprazole, respectively. The predicted pH was juxtaposed to compare the responses of both drugs.

A one-compartment model assumes a kinetically homogeneous space and first-order elimination rate kinetics. It is the simplest compartment model [17]. Model fitting (top-down approach) in a simple model could produce more robust parameters using fewer unknowns than that in complex models. In a previous study, no multi-exponential curve for the observed drug concentration could be distinguished by visual inspection after orally administering fexuprazan [3]. Here, we attempted a top-down approach with a one-compartment model to describe and predict the time-concentration profiles for fexuprazan. The model training and validation sets were adopted from the published data for two clinical trials on populations consisting of single and multiple nationalities, respectively [1,2].

PBPK models can predict drug concentrations in plasma as well as peripheral tissues (e.g., the target site) based on biologically relevant rationales. A validated PBPK model was proposed by Jeong et al. [3] for orally administered fexuprazan in humans. As the stomach is the target of fexuprazan, it is useful as an input function for pharmacodynamic models. The PBPK model was also utilized for the PPI esomeprazole [16,20].

Correlations between the area under the time-concentration curve (AUC) and gastric acidity were estimated by Sunwoo et al. (2018) and Hwang et al. (2020) [1,2]. However, they only reported the relationships between AUC, which would relate to the average concentration at steady state and the effective period (pH > 4) and could, therefore, only describe pharmacodynamics based on the static concentration. Thus, we established a pharmacokinetic model for dynamic drug concentration in the plasma and stomach and a pharmacodynamic model to clarify dose-dependent drug efficacy.

The net drug response (pH) may be the result of several biological steps rather than the direct effect of the drug on a single receptor. In past clinical trials, there was an interval between the time to maximum pH and the time to maximum plasma fexuprazan and esomeprazole concentration [1,2]. Therefore, the indirect model would be helpful to describe the dynamics of the net response using plasma fexuprazan and esomeprazole concentration (Scenarios A and B and the PK–PD model for esomeprazole) [17,18]. The delay might be caused by the fexuprazan perfusion rate to the target tissue (stomach). The fexuprazan content in the stomach was considered using the indirect and direct PD models (Scenarios C and D).

The objectives of this study were to propose pharmacokinetic–pharmacodynamic models for orally administered fexuprazan as functions of time in humans and to investigate the extended dosing method for fexuprazan based on the proposed PK–PD model’s prediction.

## 2. Materials and Methods

### 2.1. Pharmacokinetic Analysis

#### 2.1.1. Moment Analysis

The time-concentration profiles of clinical trials [1,2] were analyzed by standard moment analysis, and the pharmacokinetic parameters for fexuprazan were estimated. A non-compartmental analysis was performed with WinNonlin v. 8.1 (Pharsight Corporation, Mountain View, CA, USA) using the default options for the calculations. The linear-trapezoidal rule was used to estimate the area under the time-concentration curve (AUC) and the area under the first moment curve (AUMC). The range of the terminal phase was estimated at the number of terminal observations producing the maximum adjusted *r*^2^. The adjusted *r*^2^ was calculated as follows:

(1) $$\left(Adjusted\;r^{2}\right)=1-\frac{\left(1-r^{2}\right)\cdot \left(n-1\right)}{n-2}$$

where *n* is the number of data points and *r*^2^ is the coefficient of determination.

#### 2.1.2. One-Compartment Model

The time-concentration profiles for fexuprazan were analyzed with a one-compartment model. Fexuprazan absorption and elimination were assumed to have first-order kinetics. The following model equation was considered for the plasma fexuprazan concentration after oral administration:

(2) $$\frac{dC_{p}}{dt}=\frac{K_{a}\cdot Dose\cdot e^{-K_{a}\cdot t}-CL/F\cdot C_{p}}{V_{d}/F}$$

where $C_{p}$ and $V_{d}$ are the plasma fexuprazan concentration and the apparent volume of distribution, respectively, F is the absolute bioavailability, $K_{a}$ is the kinetic constant for absorption after oral administration, and CL is the systemic clearance. For repeated doses, the modulo operation was used for the time term at the input function ($K_{a}\cdot Dose\cdot e^{-K_{a}\cdot t}$).

The kinetic parameters were obtained by a non-linear regression at repeated doses of 20, 40, and 80 mg/day administered in clinical studies [1]. The concentration profiles were simulated using Berkeley Madonna v. 10.2.8 (Berkeley Madonna Inc., Albany, CA, USA) and the parameters obtained for the subsequent studies. The simulated and observed values [1,2] were compared against the maximum concentration at steady state (C_max,SS_) and the AUC for a dosing interval at steady state (AUC_τ_). The simulated concentrations were used in the indirect PD model at the range of 10–320 mg of fexuprazan orally administered once daily (QD) in humans (Scenario A).

#### 2.1.3. Physiologically Based Pharmacokinetic (PBPK) Model

PBPK models were used for fexuprazan and esomeprazole in the present study. The PBPK model in the previous study was utilized here for the input function of the pharmacodynamic model of fexuprazan. The authors previously developed and validated a PBPK model for PO-administered fexuprazan in humans [3]. Briefly, the extent of tissue distribution (i.e., tissue-to-plasma partition coefficients) in the PBPK model for humans was estimated using the observed partition coefficient in rats, which was adjusted after a prediction of the volume of distribution in humans using allometric scaling and an estimated volume of distribution by the Øie–Tozer equation. Simulated plasma and gastric concentrations were also used in the PBPK model to develop a pharmacodynamic model for fexuprazan. The gastric density was assumed to be 1 g/mL for the unit conversion (ng/g tissue and ng/mL) [21].

The PBPK model was also used here to describe the pharmacokinetics of orally administered esomeprazole. The population [22] was assumed to be healthy. Virtual populations were generated using the Korean healthy population files downloaded from the Simcyp repository [Compound And Population Repository. Available online: https://members.simcyp.com/account/repository/, (accessed on 16 December 2021)] [22]. The other kinetic parameters of esomeprazole were obtained from the reference file of Simcyp v. 20.0.157.0 (Certara UK Ltd., Sheffield, UK) [16]. Briefly, first-order kinetics were assumed for absorption and distribution (Kp values for tissues) and predicted based on the physicochemical and physiological properties (e.g., logP, pKa, and unbound plasma fraction) of esomeprazole [23,24]. For the validation set, time-concentration profiles were simulated with Simcyp for esomeprazole that was orally administered at 40 mg QD for 14 days. Virtual trials were performed ten times in Simcyp on five male and five female fasted subjects (100 virtual individuals) in the age range of 20–50 years old. The concentration profiles after 40 mg QD esomeprazole were obtained from the literature (Sostek et al. [20]) with the GetData Graph Digitizer v. 2.26 [GetData Graph Digitizer. Available online: http://getdata-graph-digitizer.com/, (accessed on 16 December 2021)]. The developed esomeprazole PBPK model was used to predict plasma concentration profiles, which were then used as input functions for the pharmacodynamic model.

### 2.2. Pharmacodynamic Analysis

#### 2.2.1. Indirect Response Model

An indirect response model [25] was used to describe and predict the gastric pH profiles after oral fexuprazan or esomeprazole administration. Sunwoo et al. [1] reported plasma pharmacokinetic and gastric pH pharmacodynamic profiles after oral fexuprazan administration at a 10–320 mg dose range once daily for 7 days. These values were used in the analyses of the present study. The pH profiles after 10–320 mg of fexuprazan once daily were used to determine the pharmacodynamic model parameters (*k_in_*, *I_max_*, and *IC*_50_). The estimated parameters were then applied to predict the gastric pH profiles after various dosing regimens such as twice daily of esomeprazole for anti-*Helicobacter pylori* therapy or the treatment of Zollinger–Ellison syndrome in the Republic of Korea [9].

Time-gastric pH data were obtained from the literature [1] by digitization every 30 min with the GetData Graph Digitizer v. 2.26. The data revealed that gastric pH increased at lunch, dinner, and nighttime. Therefore, the data showing pH increases in the absence of fexuprazan or esomeprazole administration were omitted. Specifically, the data for 4.5–7 h (lunch), 10.5–12.5 h (dinner), and 20–24 h (nighttime) were excluded for days 1 and 7. The duration of natural neutralization was eliminated to evaluate drug-mediated pH increases alone. The observed pH in the placebo group was used to estimate the time ranges of foods and the nocturnal effect. For the placebo group, the pH before the first burst (lunchtime) was used to calculate the mean (μ), standard deviation (σ), and Z-score [$Z=\frac{x-\mu }{\sigma }$] under the naïve condition. The pH observations at the time points when the Z-score < 1 for the observed pH in the placebo group were used to estimate the parameters for the fexuprazan and esomeprazole PD models. The initial pOH ($pOH_{inital}$) was obtained from the estimated $pH_{baseline}$. The unbound drug was assumed to be responsible for the pharmacological effect (pOH) in the equation below:

(3) $$\frac{d\mathrm{pOH}}{dt}=k_{in}\cdot \left(1-\frac{I_{max}\cdot {\left(C_{p}\cdot f_{u,p}\right)}^{\gamma }}{IC_{50}{}^{\gamma }+{\left(C_{p}\cdot f_{u,p}\right)}^{\gamma }}\right)-k_{out}\cdot \mathrm{pOH}$$

where pOH is the negative logarithmic hydroxide ion molarity. As gastric juice is aqueous, the sums of the pH and pOH were assumed to be 14 (pH + pOH = 14). *I_max_* is the fraction for maximum *k_in_* inhibition. *IC*_50_ is the unbound plasma fexuprazan concentration at 50% inhibition. The *f_u,p_* is the free plasma fexuprazan fraction (0.0645 in humans). The *k_in_* and *k_out_* are the kinetic constants of pOH. Homeostasis was assumed for gastric pH, and *k_out_* can be estimated from *k_in_* (kout=kinpOHinital) in the fexuprazan model.

#### 2.2.2. Simple Direct Effect Model

A simple direct effect model was established to compare the indirect response models used here, as the unbound concentration in the stomach tissue (the target organ) could be predicted with the PBPK model. The sigmoid *E_max_* model was proposed to describe the response of fexuprazan (altered pH). The *E_max_* model could be expressed as follows:

(4) $$\mathrm{pH}=\frac{E_{max}\times {\left(C_{stomach}\cdot f_{u,stomach}\right)}^{\gamma }}{EC_{50}{}^{\gamma }+{\left(C_{stomach}\cdot f_{u,stomach}\right)}^{\gamma }}+pH_{baseline}$$

where $E_{max}$ is the maximum attainable pH and $EC_{50}$ is the unbound gastric fexuprazan concentration at a 50% response. The Hill coefficient was incorporated as γ. The baseline pH ($pH_{baseline}$) was introduced to express the naïve gastric pH.

### 2.3. Pharmacokinetic–Pharmacodynamic Modeling

**Figure 1:**
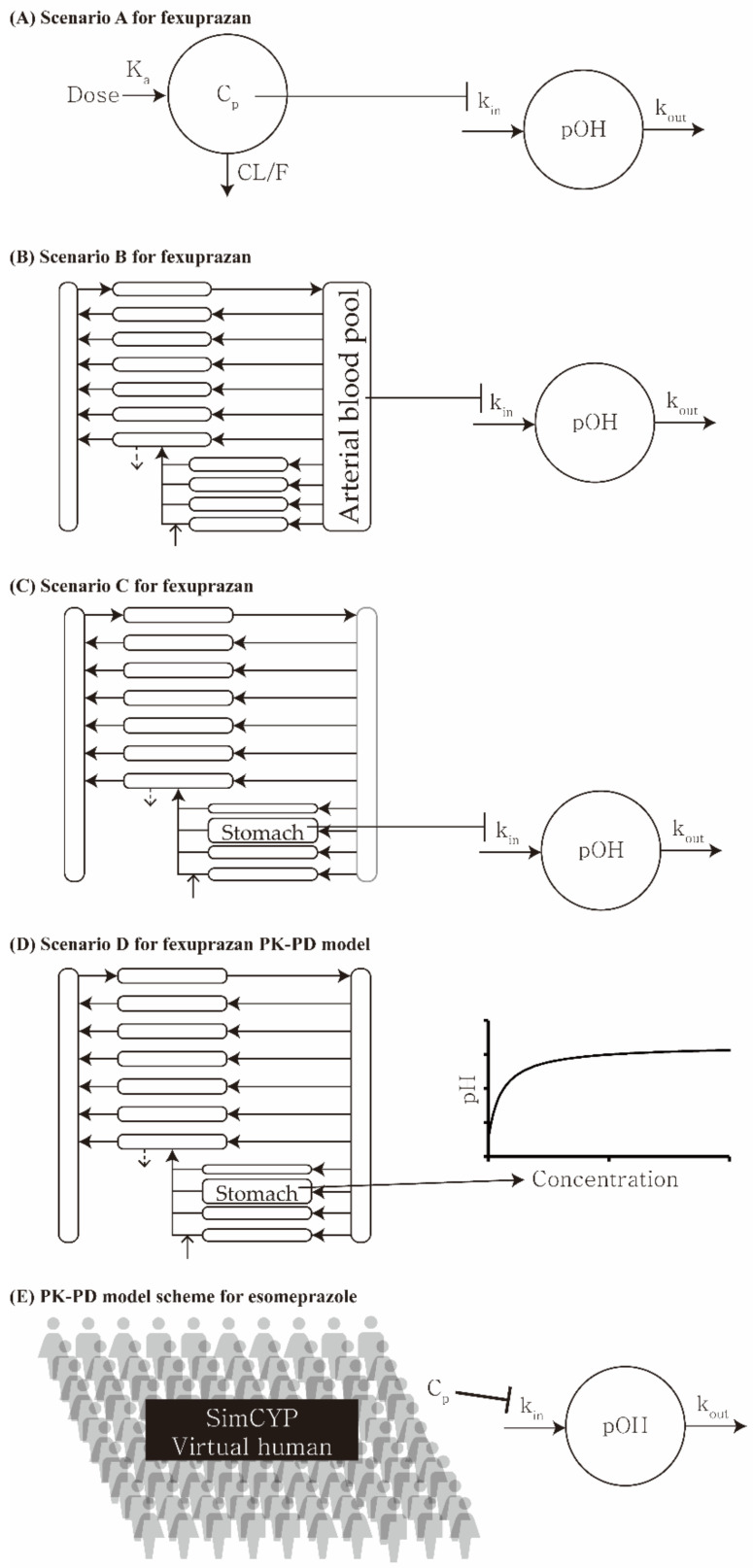
Model scheme for the PK–PD model of orally administered fexuprazan and esomeprazole in humans. Four PK–PD models were used for fexuprazan, and one PK–PD model was used for esomeprazole. The PD models for fexuprazan incorporated plasma or stomach concentration, and the one for esomeprazole used plasma concentration. Indirect models were used for the three PK–PD models for fexuprazan (Scenario A, B, and C) and the one for esomeprazole. The direct model was used for the PK–PD model scenario D.

The plasma and/or stomach concentrations were used in the pharmacokinetic analysis. The indirect effect and simple direct effect models were used in the pharmacodynamics analysis. The pharmacokinetic–pharmacodynamic model was considered using Scenarios A, B, C, and D (Table 1 and Figure 1). Scenario A is the plasma concentration simulated by the one-compartment and indirect response models. Scenario B is the plasma concentration simulated by the PBPK and indirect response models. Scenario C is the stomach concentration simulated by the PBPK and indirect response models. Scenario D is the stomach concentration simulated by the PBPK and simple effect models.

The unbound gastric concentration was estimated using the PBPK model and was the product of the total gastric concentration and the free fexuprazan fraction in the stomach ($C_{stomach}\cdot f_{u,stomach}$). The free fexuprazan fraction in the stomach was calculated using the stomach-to-plasma concentration ratio and the free plasma fraction (fu,stomach=fu,pKp,stomach; 0.000335 in humans). The model parameters were obtained using the Curve Fit function in Berkeley Madonna v. 10.2.8.

The PK–PD model for esomeprazole incorporated the unbound plasma concentration from the Simcyp PBPK model. The observed pH was digitized from the published data for 40 mg QD oral esomeprazole administration in the Korean population [1] and the GetData Graph Digitizer. The indirect model was adapted as the PD model for esomeprazole using the Simcyp PBPK model. The PD model response was pOH, which was converted to pH by the same method used for the fexuprazan model (pH + pOH = 14). The PD model parameters were estimated using the Parameter Estimation function in Simcyp. The Nelder–Mead method was used to minimize the least squares. The Simcyp simulator could estimate the plasma esomeprazole concentration distribution for the virtual population. Hence, the 90% interval could be estimated using the 90% range of simulated concentrations. No distribution was assumed for the PD model parameters.

### 2.4. Statistics

Unless otherwise specified, the estimated parameters were presented as the means ± standard deviation (SD). Unless otherwise specified, the SD for the in vivo experiments were presented as inter-individual variabilities. When skewed distributions were observed (fold differences), the median (5th and 95th percentiles) was presented. The fold difference (fold difference=yi^yi), root mean squared error (*RMSE*), root mean squared logarithmic error (*RMSLE*), and R-squared (*r*^2^) were calculated for the PK–PD models. The RMSLE could be estimated using the following equation [26]:

(5) $$RMSLE=\sqrt{\frac{1}{n}\cdot \overset{n}{\underset{i=1}{\sum }}{\left\{\ln\left(y_{i}\right)-\ln\left({\hat{y}}_{i}\right)\right\}}^{2}}$$

where *n* is the number of data and $y_{i}$ and ${\hat{y}}_{i}$ are the observed and simulated pH values. The *RMSE* was estimated using the following equation [26]:

(6) $$RMSE=\sqrt{\frac{1}{n}\cdot \overset{n}{\underset{i=1}{\sum }}{\left(y_{i}-{\hat{y}}_{i}\right)}^{2}}$$

The *r*^2^s were calculated for the PK–PD models using the RSQ function in Microsoft Excel v. 2110 build 16.0.14527.20276 (Microsoft Corporation, Redmond, WA, USA).

## 3. Results

### 3.1. Pharmacokinetic Analysis

Data for the 20, 40, and 80 mg/day doses were used for the training set, while data for the 40 and 80 mg/day doses were used for the validation set for the one-compartment model. The previously developed PBPK model was used as a reference model. The training set consisted of time-concentration profiles after 7 days of fexuprazan doses, full pharmacokinetic profiles for days 1 and 7, and trough concentrations between days. The validation set consisted of the profiles after 8 days of fexuprazan doses, full pharmacokinetic profiles for days 1 and 8, and trough concentrations between doses. The pharmacokinetic parameters for fexuprazan were estimated via non-compartmental analysis. The results of the latter were assumed to be equivalent to those for the control in the one-compartment model. Briefly, the apparent clearances at steady state (CL_SS/F_) were 1810 ± 988 mL/min and 1910 ± 862 mL/min for the training [1] and validation sets [2], respectively. The areas under the curve over the dosing interval at steady state (AUC_τ_) were 1.36 × 10^4^ ± 9.65 × 10^3^, 2.51 × 10^4^ ± 6.12 × 10^3^, and 5.91 × 10^4^ ± 2.08 × 10^4^ min·ng/mL for the 20, 40, and 80 mg dose groups in the training set, respectively, and 2.34 × 10^4^ ± 1.16 × 10^4^ and 5.18 × 10^4^ ± 1.87 × 10^4^ min·ng/mL for the 40 and 80 mg dose groups in the validation set, respectively. The one-compartment model was applied to describe the fexuprazan kinetics after oral administration. The maximum concentrations after oral fexuprazan administration (C_max,SS_) were 20.8 ± 14.4, 43.2 ± 11.6, and 94.4 ± 36.5 ng/mL for the 20, 40, and 80 mg fexuprazan doses in the training set, respectively, and 35.5 ± 19.3 and 78.9 ± 34.0 ng/mL for the 40 and 80 mg fexuprazan doses in the validation set, respectively.

**Figure 2:**
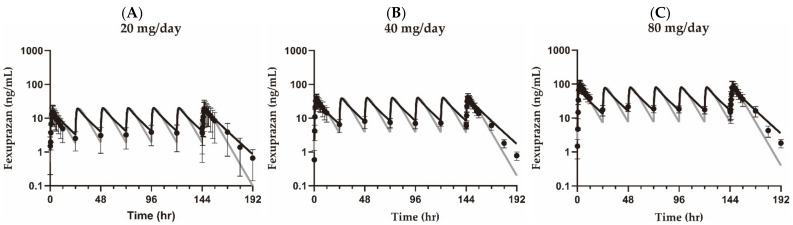
Pharmacokinetic profiles of fexuprazan in the training set for the one-compartment model. Observed data and simulated lines are plotted as markers and lines after 20 (**A**), 40 (**B**), and 80 mg/day (**C**) dosing. Closed circles (●) represent the mean and standard deviation of observed fexuprazan concentrations in plasma after PO administration. Black lines represent simulated plasma fexuprazan concentrations using the PBPK model, and gray lines are fitted concentrations using the one-compartment model.

The kinetic constant for the absorption (K_a_), the volume of distribution (V_d_/F), and the apparent clearance (CL/F) of the one-compartment model were estimated to be 0.00928 min^−1^, 736,000 mL, and 1510 mL/min, respectively. Visual inspection and comparison were made between the observed and simulated data in the one-compartment model (Figure 2). The PK parameters were compared against the model-predicted parameters for the 20, 40, and 80 mg/day fexuprazan doses (Table 2). The simulated AUC_τ_ values were 1.32 × 10^4^, 2.65 × 10^4^, and 5.30 × 10^4^ min·ng/mL for the 20, 40, and 80 mg fexuprazan doses, respectively, using the one-compartment model and 1.49 × 10^4^, 3.00 × 10^4^, and 6.04 × 10^4^ min·ng/mL for the 20, 40, and 80 mg fexuprazan doses, respectively, using the PBPK model. The simulated C_max,SS_ values were 19.0, 37.9, and 75.9 ng/mL for the 20, 40, and 80 mg fexuprazan doses, respectively, using the one-compartment model and 20.2, 40.4, and 81.2 ng/mL for the 20, 40, and 80 mg PO fexuprazan doses, respectively, using the PBPK model. The compartment model could predict the kinetic parameters in a two-fold range (AUC_τ_ and C_max_). The fold differences between the simulated and observed values for AUC_τ_ and C_max,SS_ were 0.883 to 0.977 and 0.961 to 1.07, respectively, in the validation set. The predicted values from the PBPK model yielded values comparable to those from the one-compartment model (Table 2 and Figure 2). Moreover, the PBPK model for esomeprazole was evaluated. The predicted median C_max_ and CL/F were 1180 ng/mL and 19.7 L/h, respectively, in a two-fold range. In contrast, the literature values were 1220 ng/mL and 14.6 L/h, respectively [20].

### 3.2. Pharmacodynamic Models

**Figure 3:**
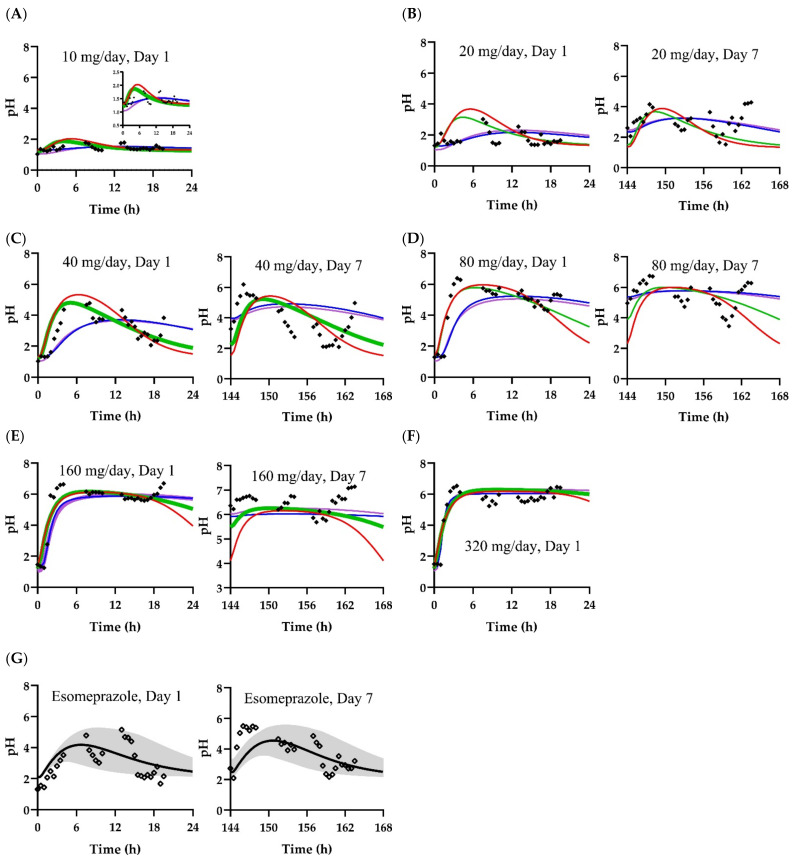
Gastric pH after oral fexuprazan and esomeprazole administration. Closed diamonds (◆) represent observed pH after the oral administration of 10 (**A**), 20 (**B**), 40 (**C**), 80 (**D**), 160 (**E**), and 320 mg fexuprazan QD (**F**). Open diamonds (◇) represent observed pH after the administration of 40 mg QD esomeprazole (**G**). Red and green lines represent simulated pH profiles for the PK–PD model using unbound plasma concentrations in the one-compartment (Scenario A) and PBPK (Scenario B) models, respectively. Blue and purple lines are simulated pH using the indirect (Scenario C) and direct PD (Scenario D) models, respectively. Black lines are the medians of simulated pH among the virtual population through the indirect model incorporating unbound plasma esomeprazole concentration in the PBPK model. Gray shadow represents the 90% range of the simulated pH for esomeprazole.

**Figure 4:**
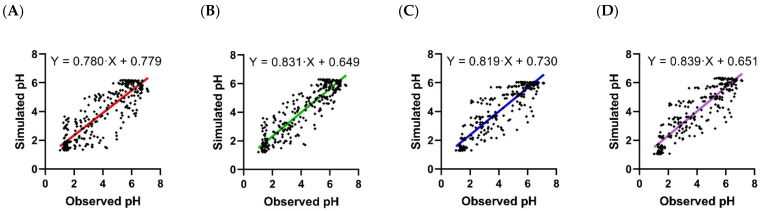
Comparison between observed and simulated pH using four PK–PD models for fexuprazan. X- and Y-axes represent observed and simulated pH, respectively, after 10, 20, 40, 80, 160, and 320 mg/day. One-compartment–PD (**A**), PBPK–PD (**B**), stomach-indirect–PD (**C**), and stomach-direct–PD (**D**) models estimated simulated pH. Closed diamonds (◆) represent observed and simulated pH at drug effect time points. Red (**A**), green (**B**), blue (**C**), and purple (**D**) lines are the results of the linear regression of markers. *r*^2^s are 0.738, 0.827, 0.803, and 0.808 for red, green, blue, and purple lines, respectively.

The parameters of the PD models for fexuprazan (Scenarios A, B, C, and D) were estimated using the one-compartment and PBPK models separately as input functions (Figure 3). The estimated parameters for the PD models are listed in Table 3. Briefly, for the PD model using the plasma fexuprazan concentrations of the one-compartment (Scenario A) and the PBPK (Scenario B) models as input functions, the *k_in_* values were 0.132 and 0.139, the *I_max_* values were 0.386 and 0.401, and the *IC*_50_ values were 1.06 and 1.16 ng/mL, respectively. For the indirect model using the gastric fexuprazan concentration as the input function (Scenario C), the *k_in_*, *I_max_*, and *IC*_50_ values were 1760, 0.378, and 0.919 ng/mL, respectively. For Scenario D (the direct PD model using the gastric fexuprazan concentration), the *pH_baseline_*, *E_max_*, *γ*, and *EC*_50_ values were 1.04, 5.50, 1.58, and 0.992 ng/g tissue, respectively. The *IC*_50_ and *EC*_50_ values were the unbound fexuprazan concentrations in the plasma or stomach. The estimated *RMSLE*, *RMSE*, and *r*^2^ values were 0.315, 0.992, and 0.738 for Scenario A, 0.262, 0.800, and 0.827 for Scenario B, 0.243, 0.853, and 0.803 for Scenario C, and 0.249, 0.845, and 0.808 for Scenario D based on the data for the 10–320 mg fexuprazan/day (Table 4) dosage. The best-fitting models among the four PK–PD scenarios differed with the criteria. Scenario C could be selected based on the RMSLE, whereas Scenario B could be selected based on the *RMSE* and *r*^2^. Considering the y-intercept and the slope with *r*^2^s, however, Scenario D was apparently the best among the four PK–PD models (Figure 4). The observed and predicted pH after 10, 20, 40, 80, 160, and 320 mg QD fexuprazan doses are plotted in Figure 3. Among the observed and simulated pH, the 93.4, 96.2, 98.3, and 98.3% data points were in the range of the two-fold difference or the >0.5 and <2× difference for Scenarios A, B, C, and D, respectively. The ranges of the fold differences from the 5th to 95th percentiles were 0.572–1.64, 0.688–1.67, 0.691–1.59, and 0.696–1.62-fold for Scenarios A, B, C, and D, respectively. Scenario C had the narrowest 5th to 95th percentile range in the fold difference. (Table 4). The observed and simulated pH values were plotted for the four models (Figure 4).

The parameters of the PK–PD model for esomeprazole were fitted to the observed pH. The ‘simple Emax model’ and ‘first order from SS’ were selected as the Response Model and Parameterized Link Model, respectively, in Simcyp to apply the indirect model. The model parameters were 2.25 pOH/h, 0.189/h, and 0.0388 μM for *k_in_*, *k_out_*, and *IC*_50_, respectively. *I_max_* was fixed as 0.5 (not fitted). The time-pH profiles were simulated after esomeprazole was orally administered at 40 mg/day (Figure 3G,H). The *RMSLE* and *RMSE* were 0.269 and 0.873, respectively, for the PK–PD model of esomeprazole.

## 4. Discussion

Fexuprazan is a potassium-competitive acid blocker (P-CAB), a relatively new drug class compared with proton pump inhibitors (PPIs). Despite the differences in their H^+^/K^+^ ATPase binding mechanisms (reversible or irreversible inhibition), their clinical effects are similar in that they both increase gastric pH. The PPI esomeprazole is usually administered once daily. Nevertheless, its package insert also includes 20 mg BID and 40 mg BID regimens to treat *H. pylori* infection and Zollinger–Ellison syndrome, respectively [9]. For the P-CAB tegoprazan, there was a 50 mg BID regimen to treat *H. pylori*, but there was none for Zollinger–Ellison syndrome. Tegoprazan was one of the first P-CABs approved in the Republic of Korea [27]. Registered clinical trials (NCT04341454, NCT04490434, and NCT03487562 at clinicaltrials.gov (accessed on 16 December 2021)) on fexuprazan BID dosing are in progress. For ethical and economic reasons, the clinical trials may not include every possible dosing schedule. In the present study, several PK–PD models were proposed to predict gastric pH after oral fexuprazan administration [1,2]. If the PK model had been refined after intravenous administration, it would have been possible to predict the pH after intravenous bolus doses based on PK–PD modeling. The circadian pattern was observed in the baseline pH, which made deviation of the model’s prediction from the observed pH. The pharmacodynamic models here did not incorporate the rhythm of proton secretion [28,29,30]. However, the PK–PD model is still useful for comparing the effects of the two drugs and for proposing a new regimen for fexuprazan in humans.

Here, the one-compartment model was proposed for orally administered fexuprazan in humans. It was attempted because of the apparently monophasic and linear kinetics of fexuprazan in terms of the volume of distribution, systemic clearance, and absolute bioavailability in the PBPK models of previous studies. According to C_max_ and AUC_τ_, the model described or predicted time-concentration profiles in a two-fold range for 20, 40, and 80 mg/day fexuprazan in humans. There were no observed concentration profiles following intravenous administration in humans. Thus, the apparent volume of distribution (V_d_/F) and clearance (CL/F) were estimated as model parameters. The estimated clearances were comparable between the non-compartmental and compartmental analyses (CL_SS_/F and CL/F).

The compartment and PBPK models were used to predict plasma fexuprazan concentrations as input functions for the indirect PD model (Scenarios A and B). The simulated pH using the plasma fexuprazan concentration in the PBPK model (Scenario B) fit better than the one-compartment model (Scenario A) based on the range of fold difference, *RMSE*, *RMSLE*, and *r*^2^. Compared with the PBPK model [3], the terminal phase concentration was underpredicted by the one-compartment model (Figure 2). However, the deviation was negligible in terms of AUC_τ_ (Table 2). Visual inspection implied that the PK–PD models differed in terms of their pH predictions for the terminal phase (Figure 3). The observed differences between the predictions from the PK–PD models may have been the result of superior prediction by the PBPK model at low fexuprazan concentrations.

The indirect and direct PD models were attempted using the gastric fexuprazan concentration in the PBPK model (Scenarios C and D). The stomach was taken to be the target tissue of fexuprazan. The indirect model was attempted using the predicted gastric fexuprazan concentration (Scenario C). The PK–PD model for Scenario C fits better than that for Scenario A in terms of fold difference, *RMSE*, *RMSLE*, and *r*^2^, but it fits worse than that for Scenario B in terms of *RMSE* and *r*^2^. Scenario C showed higher *k_in_* than the other indirect PK–PD models (Scenarios A and B). The calculated mean transit times (MTTs) for the stomach and blood were 682 and 1.00 min, respectively (c.f., for the small molecule drug, the mean transit time would be calculated as $MTT_{T}=V_{T}\times K_{p}/\left(Q_{T}\times R_{b}\times f_{d}\right)$; $MTT_{T}$: Mean transit time for the tissue; $V_{T}$: Anatomical tissue volume; $Q_{T}$: Blood flow of the tissue; $K_{p}$: Tissue-to-plasma partition coefficient; $R_{b}$: Blood-to-plasma partition coefficient; $f_{d}$: Fraction of distribution, which would be 1 for the well-stirred compartment), and the discrepancy might contribute to part of the delay from the concentration in plasma to the effect in the stomach. As the stomach is thought to be the target organ for P-CABs [2,13,31,32], the high kin value seemed to be reasonable. The direct PD model was also attempted using the gastric fexuprazan concentration (Scenario D). Scenario D showed a lower *RMSE* than Scenario C, and its *RMSLE*, fold difference range, and *r*^2^ were comparable to those for Scenario C. The PK–PD model for Scenario D showed lower biased prediction than that of Scenario C in terms of slope, y-intercept, and *r*^2^ (Figure 4). The comparison among Scenarios B, C, and D would imply the importance of fexuprazan kinetics in the stomach, which is assumed to be the target organ of this drug. The estimated *IC*_50_s and EC_50_ among the four PK–PD models were comparable to the observed *IC*_50_ in vitro, which were 25 and 26 nM (10.3 and 10.7 ng/mL in the media; c.f., molar mass: 410.4 g/mol) against H^+^/K^+^ ATPase at pH values of 7.4 and 6.4, respectively, despite the systemic differences (in vitro vs. in vivo) [33].

**Figure 5:**
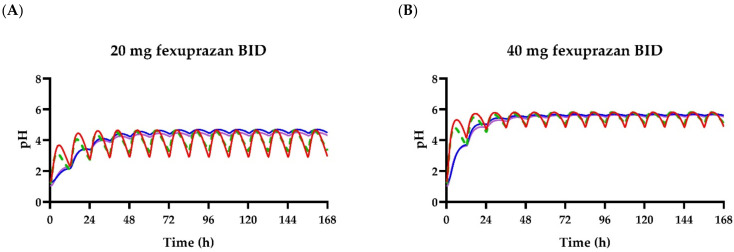

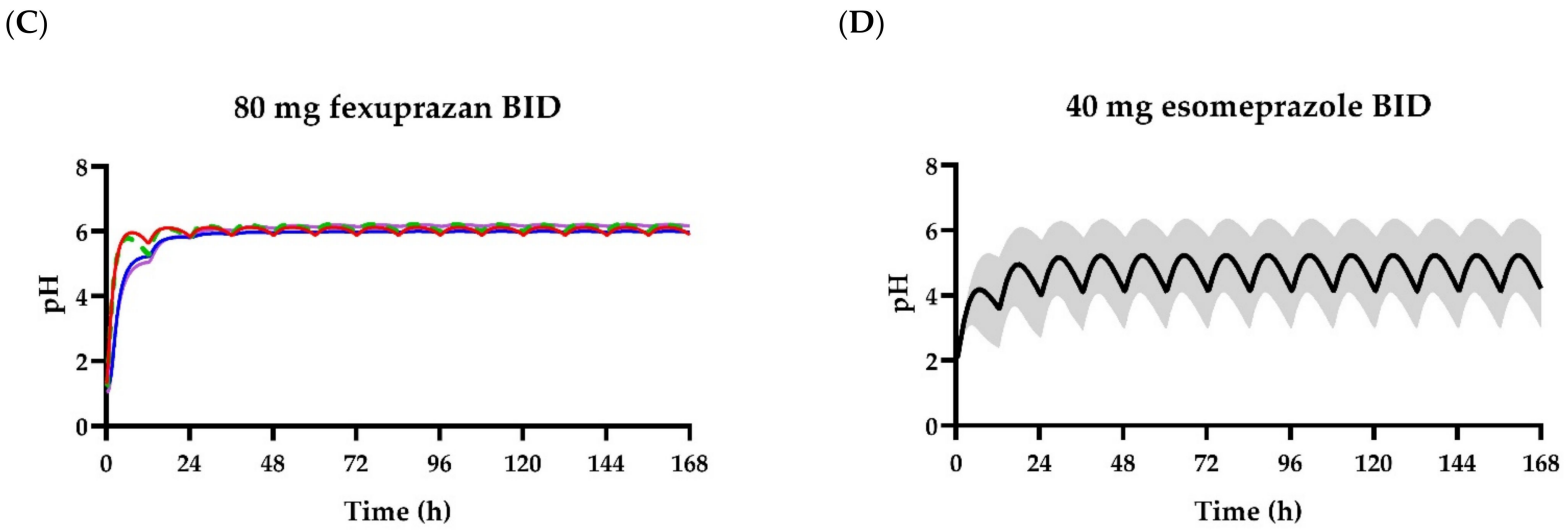
Simulated intragastric pH profiles for fexuprazan and esomeprazole BID. Time-pH profiles are shown after fexuprazan 20 mg BID (**A**), 40 mg BID (**B**), and 80 mg BID (**C**), as well as esomeprazole 40 mg BID (**D**). Red, green (dashed), blue, and purple lines represent Scenarios A, B, C, and D for fexuprazan, respectively (see text). The black solid line is the median of simulated pH after the oral administration of esomeprazole (40 mg BID) in the virtual population. Gray shadow is the 90% range of the predicted pH.

The 90% interval encompassed most of the observations in the indirect model for esomeprazole (Figure 3G). The *RMSLE* and *RMSE* for the esomeprazole PK–PD model were comparable to those for the model of Scenario B for fexuprazan, which consisted of the plasma concentrations from the PBPK and indirect PD models. As the fexuprazan doses had a wider range (10, 20, 40, 80, 160, and 320 mg/d) than the esomeprazole doses (40 mg/day), the number of observed pH values for fexuprazan was larger than that for esomeprazole. As the *r*^2^ was affected by the number and range of data [34,35], no comparison could be drawn between the PK–PD models of fexuprazan and esomeprazole using *r*^2^. As previously reported [1], the model estimations exhibited similar acid suppression profiles in humans at 40 mg/day for both fexuprazan and esomeprazole (Figure 3). Fexuprazan at 40 mg BID would theoretically have a similar efficacy to the same dose of esomeprazole in terms of regulating gastric pH (Figure 5).

The PK–PD model could estimate pH, and over 95% of the observed pH values were predicted within a two-fold range by the proposed PBPK–PD models. Thus, 96.2, 98.3, and 98.3% of the predicted pH values were within the two-fold range for Scenarios B, C, and D, respectively. Nonetheless, there was a discrepancy between the observed and simulated pH values, possibly because of the structure of the PBPK model for fexuprazan. This model assumed first-order fexuprazan absorption directly into the portal vein rather than the stomach and large and small intestine compartments. This assumption limited the fexuprazan distribution under gastric blood flow. The absorption kinetics in the PBPK model should be refined to predict the gastric fexuprazan concentration more accurately. Furthermore, the gastric absorption kinetics could be indirectly adjusted using the observed bioavailability or the pH observations made following the intravenous administration of fexuprazan. However, these are not yet applicable. Circadian rhythms and foods can elevate gastric pH [28,29,30]. However, these effects were not integrated into the PD model. The pH observations for the placebo group distinguished pH increases not induced by drugs. Therefore, the PD model could estimate the expected drug effect on an empty stomach.

The pH profiles were predicted using the PK–PD models after the administration of fexuprazan at doses of 20, 40, and 80 mg QD and 40 mg BID. Insights can be drawn from the PK–PD model-based predictions despite the fact that there were slight differences among the four PK–PD models. Nocturnal gastric pH could be reduced to <4 by administering 80 mg QD fexuprazan (observed pH) but could nonetheless be improved by administering 40 mg BID fexuprazan (predicted pH) according to the PK–PD model predictions (Figure 3 and Figure 5). Both the drug response and regimen would affect compliance. Complex regimens tend to decrease drug compliance [36,37]. The QD regimen for fexuprazan could be changed to BID to improve drug compliance in the combination therapy (amoxicillin and clarithromycin BID) prescribed for *H. pylori* infection. In fact, BID regimens have been recommended for approved PPIs, such as esomeprazole and lansoprazole, as well as for P-CABs such as tegoprazan, which is prescribed for the treatment of *H. pylori* [9,27,38].

## 5. Conclusions

In the present study, PK–PD models were developed by measured gastric pH in humans orally administered with fexuprazan at doses ranging from 10 to 320 mg/day. In future research, the preceding PD model can be adapted to the fexuprazan concentration predicted by more refined PK models, including the proportion of drug-metabolizing enzymes, the absolute bioavailability, and the absorption in the human intestine. Future PBPK models could also integrate the effects of perpetrator drugs and the physiological parameters of elderly and pediatric populations. The impacts of drug–drug interactions and physiology could be directly linked to the effects of fexuprazan and could be used in the rational design of dosing regimens suited for actual clinical conditions. The doses and dosing intervals may be adjusted, and new formulations can be developed.

## Figures and Tables

**Table 1:** Four PK–PD modeling scenarios for orally administered fexuprazan. The PK models are one-compartment and PBPK, while the PD models are indirect response and simple direct effect.

Scenario	Pharmacokinetic Model	Pharmacodynamic Model
A	One-compartment (plasma)	Indirect response
B	PBPK (plasma)	Indirect response
C	PBPK (stomach)	Indirect response
D	PBPK (stomach)	Simple direct effect

**Table 2:** Estimated kinetic parameters for orally administered fexuprazan in humans. Simulated AUC_τ_ and C_max,SS_ were in a two-fold range of observed values for the training and validation sets.

Parameter (Unit)	Group		20 mg QD	40 mg QD	80 mg QD
AUC_τ_ (min × ng/mL)	Observed	Training set	1.36 × 10^4^ ± 9.65 × 10^3^	2.51 × 10^4^ ± 6.12 × 10^3^	5.91 × 10^4^ ± 2.08 × 10^4^
		Validation set	-	2.34 × 10^4^ ± 1.16 × 10^4^	5.18 × 10^4^ ± 1.87 × 10^4^
	Simulated	Compartment model	1.32 × 10^4^	2.65 × 10^4^	5.30 × 10^4^
		PBPK model	1.49 × 10^4^	3.00 × 10^4^	6.04 × 10^4^
	AUC_ratio_	Compartment model	1.03	0.947	1.12
			-	0.883	0.977
		PBPK model	0.913	0.837	0.978
			-	0.780	0.858
C_max,SS_ (ng/mL)	Observed	Training set	20.8 ± 14.4	43.2 ± 11.6	94.4 ± 36.5
		Validation set	-	35.5 ± 19.3	78.9 ± 34.0
	Simulated	Compartment model	19.0	37.9	75.9
		PBPK model	20.2	40.4	81.2
	C_max,ratio_	Compartment model	1.09	1.14	1.24
			-	0.937	1.04
		PBPK model	1.03	1.07	1.16
			-	0.878	0.972

**Table 3:** Estimated parameters for the fexuprazan PD models. Four combinations of PK–PD models were attempted. Unbound concentrations in plasma (Scenarios A and B) and stomach (Scenarios C and D) were used as input functions. The PK–PD models were designated Scenarios A, B, C, and D. Detailed descriptions of the PK–PD models are described in the text.

Scenario	Parameter	Value
A	*pH_baseline_*	1.30
	*k_in_* (pOH/min)	0.132
	*I_max_* (ratio)	0.386
	*IC*_50_ (ng/mL)	1.06
	*γ*	2.51
B	*pH_baseline_*	1.22
	*k_in_* (pOH/min)	0.139
	*I_max_* (ratio)	0.401
	*IC*_50_ (ng/mL)	1.16
	*γ*	2.14
C	*pH_baseline_*	1.29
	*k_in_* (pOH/min)	1760
	*I_max_* (ratio)	0.378
	*IC*_50_ (ng/g tissue)	0.919
	*γ*	2.16
D	*pH_baseline_*	1.04
	*E_max_* (pH)	5.50
	γ	1.58
	*EC*_50_ (ng/g tissue)	0.992

**Table 4:** Statistics for the four scenarios. Smaller RMSLE and RMSE values would imply a better fitting to the observations. In the case of the fold difference and *r*^2^, the model *r*^2^ values closer to 1 have better description power for the observed data. The fold differences are presented with the medians and the 5th to 95th percentiles.

Scenario	Scenario A	Scenario B	Scenario C	Scenario D
Fold difference	1.00 (0.572, 1.64)	0996 (0.688, 1.67)	0.992 (0.691, 1.59)	0.988 (0.696, 1.62)
*RMSLE*	0.315	0.262	0.243	0.249
*RMSE*	0.992	0.800	0.853	0.845
*r* ^2^	0.738	0.827	0.803	0.808

## Data Availability

Data is contained within the article.
